# Combined treatment with naringin and osthole ameliorates colitis through microbiota–amino acid metabolism and the JNK pathway

**DOI:** 10.1007/s13659-025-00582-z

**Published:** 2026-02-04

**Authors:** Mengqin Chen, Zihao Lu, Tong Zhang, Guoping Li, Qingyu Zheng, Tao Zhang

**Affiliations:** College of Veterinary Medicine, Anhui Agricultural University, Hefei, 230031 China

**Keywords:** Naringin, Osthole, Intestinal barrier, Ulcerative colitis, Gut microbiota, Amino acid metabolism

## Abstract

Inflammatory bowel disease (IBD), particularly ulcerative colitis, involves disruption of the intestinal mucosal barrier due to ecological and metabolic imbalances in the gut as its underlying pathology. Current therapies for Ulcerative colitis (UC) exhibit limited efficacy and adverse effects, necessitating the development of novel treatment strategies. Naringin and osthole are natural herbal compounds that show therapeutic potential in various inflammatory models due to their excellent anti-inflammatory activity. However, their combined therapeutic effects and precise mechanisms in UC remain unreported. This study aimed to explore the therapeutic effectiveness and mechanism of naringin combined with osthole in addressing dextran sodium sulfate (DSS)-induced colitis. The investigation centered on their impact on the disruption of the intestinal epithelial cell barrier, modulation of intestinal flora composition, alteration of metabolites, and inflammation model in vitro. Modal assessment encompassed body weight, disease activity index (DAI) score, colon length, and histopathological examination. Intestinal barrier integrity was evaluated through Quantitative Real-Time PCR, western blotting, and immunofluorescence staining. Microbiota abundance and metabolic levels were assessed using 16S ribosomal RNA gene sequencing and metabolomics analysis. Protein expression levels of pertinent pathways and associated receptors were tested through network pharmacology prediction and western blot analysis. Naringin and osthole synergistically relieved colitis symptoms in mice compared with either drug alone or 5-aminosalicylic acid, as evidenced by weight loss recovery, DAI scores, and colon length preservation. Mechanistically, naringin combined with osthole down-regulated the expression level of JNK/NF-κB signaling pathway related proteins and repaired intestinal barrier. Furthermore, the combination regulates the composition of the microflora and promotes the restoration of a steady state of the microflora. Metabolomic revealed amino acid-tryptophan metabolism as a key metabolic pathway. It also reveals the microbiota-tryptophan pathway as a potential therapeutic strategy. Naringin combined with osthole can alleviate DSS-induced colitis more effectively by JNK/NF-κB signaling pathway, repairing barrier function and regulating intestinal microbiota and metabolites. These findings provide a theoretical basis for the combination therapy strategy to enhance the efficacy of potential functional food in treating ulcerative colitis.

## Introduction

Ulcerative colitis (UC) serves as a quintessential model of inflammatory bowel disease (IBD), presenting as a persistent inflammatory state in the intestinal mucosa [1]. The etiology of UC is multifaceted, involving a complex interplay of psychological, environmental and various factors leading to intestinal barrier compromise and immune dysregulation, culminating in localized mucosal damage[2].

UC is characterized by chronicity and high propensity for recurrence. The primary approach to managing UC revolves around mitigating inflammation over the longterm [3]. Current therapeutic options encompass immunosuppressants, aminosalicylates and corticosteroids, however, less than half of patients achieve sustained remission, often necessitating colectomy [4]. The unpredictable clinical trajectory of UC, marked by alternating periods of exacerbation and remission, has fueled its increasing global prevalence [1]. Hence, there is an imperative to develop treatments for colitis that offer minimal adverse effects and target colonic inflammation with precision.

Numerous studies have established a robust association between gut microbiota equilibrium and colitis [5, 6]. Inflammatory processes and immune reactions dysregulation have been shown to disrupt the gastrointestinal environment, leading to gut microbiota dysbiosis and mucosal barrier impairment [7, 8]. Reports indicate that diminished microbial diversity and reduced Firmicutes abundance are prominent features in individuals with inflammatory bowel disease (IBD) [9]. Intestinal permeability plays a crucial role in modulating inflammatory gene expression, with alterations in pathogenic bacteria contributing to its changes [9]. The reshaping of the intestinal microbiota has been linked to the suppression of tight junction proteins in the intestinal epithelial barrier, consequently elevating intestinal mucosal permeability [10, 11]. The microbial community migrates to the lamina propria, inducing the release of pro-inflammatory factors like interleukin-6 (IL-6) and interleukin-1 (IL-1β), thereby driving immune cell recruitment. The involvement of gut microbiota in metabolic disorder development in various animal models via microbial metabolites has been established [12]. For example, the accumulation of substrates by gut microbiota, such as carbohydrates and oligosaccharides, results in the production of short-chain fatty acids (SCFA), a crucial metabolite influencing inflammation, intestinal barrier function and oxidative stress [13, 14]. Therefore, current therapeutic strategies for colitis primarily target restoring the intestinal barrier, alongside modulating gut microbiota and metabolites.

Traditional Chinese medicine offers several advantages, such as safety, affordability, and efficacy in treating intestinal inflammation [15]. Previous studies have highlighted the role of naringin in mitigating uterine inflammation [16]. Osthole (7-methoxy-8-(3-methylbut-2-enyl) chromen-2-one, PubChem CID:10228, CAS No: 484-12-8), the primary active compound in *Cnidium monnieri* (L.) Cuss, is a natural coumarin with documented anti-proliferative, anti-inflammatory, and apoptosis inducing properties, suggesting its potential therapeutic value in inflammatory conditions like arthritis, colitis, and pancreatitis [17–19]. While various studies have explored the mechanisms through which traditional Chinese herbs alleviate DSS-induced colitis-such as turmeric's inhibition of the NF-κB pathway to reduce intestinal inflammation and Zuojin Pill's regulation of the MAPK pathway to maintain mucosal integrity—limited research exists on the specific therapeutic mechanisms of naringin alone or in conjunction with osthole in treating DSS-induced colitis [15, 20].

In this investigation, we effectively induced DSS-induced colitis in mice following the methodology outlined by Ma et al. [21]. Through a synthesis of network pharmacology and previous studies on osthole in different disease models, we confirmed the individual inhibitory effects of naringin and osthole on DSS-induced colitis in mice by regulating the JNK/NF-κB pathway and impacting the balance of gut microbiota and metabolites. Additionally, this research explores the potential enhanced therapeutic benefits of combination therapy compared to monotherapy.

## Materials and methods

### Reagents and antibodies

Dextran sulfate sodium (DSS) (#MB5535-1) was sourced from MeilunBio® (Dalian, China). Naringin (purity over 98%, CAS#: T21431-1 g) and osthole (HPLC ≥ 98%, CAS#:484-12-8) were bought from Shanghai Yuanye Bio-Technology Co. Ltd. (Shanghai, China). 5-Aminosalicylic acid (5-ASA) (HY-15,027, MedChemExpress, USA) was first dissolved in DMSO to create a stock solution, which was subsequently diluted with corn oil to achieve a final concentration of 10 mg/mL, the final concentration of DMSO was less than 0.1%. Lipopolysaccharide (LPS) (E. coli 055: B5) was provided by Sigma-Aldrich (St. Louis, Missouri, USA). The JNK inhibitor (SP600125) was obtained from Targeted Mol (USA). Dulbecco's Modified Eagle Medium (DMEM), penicillin–streptomycin, and fetal bovine serum (FBS) were purchased from Gibco (USA). Further details regarding the antibodies can be found in Table 1.

**Table 1:** Information on antibodies used in the study

Antibodies	Product code	Dilution	Species	Manufacturer
GAPDH	BL072A	1: 1000 (WB)	Mouse	Biosharp
Anti-mouse IgG	BL001A	1:50,000 (WB)	Goat	Biosharp
Anti-rabbit IgG	RS0002	1:20,000 (WB)	Rabbit	Immunoway
p-NF-kb p65	sc-136548	1:1000 (WB)	Mouse	Santa Cruz Biotechnology
NF-kb p65	sc-8008	1:1000 (WB)	Mouse	Santa Cruz Biotechnology
p-ikk	sc-293135	1:1000 (WB)	Mouse	Santa Cruz Biotechnology
ikk	sc-7606	1:1000 (WB)	Mouse	Santa Cruz Biotechnology
p-ikb	sc-8404	1:1000 (WB)	Mouse	Santa Cruz Biotechnology
ikb	sc-1643	1:1000 (WB)	Mouse	Santa Cruz Biotechnology
p-p38	PC5476	1:2000 (WB)	Rabbit	Abmart
p38	T55600	1:2000 (WB)	Rabbit	Abmart
β-Actin	T40104	1:10,000 (WB)	Mouse	Abmart
p-ERK	AF1015	1:2000 (WB)	Rabbit	Affinity
ERK	AF0155	1:5000 (WB)	Rabbit	Affinity
Bcl-2	AF6139	1:1000 (WB)	Rabbit	Affinity
Cleaved-Caspase-3	AF7022	1:2000 (WB)	Rabbit	Affinity
p-JNK	80024-1-RR	1:4000 (WB)	Rabbit	Proteintech
JNK	66210-1-Ig	1:20,000 (WB)	Mouse	Proteintech
Bax	50599-2-Ig	1:2000 (WB)	Rabbit	Proteintech
Caspase-3	9661 T	1:1000 (WB)	Rabbit	CST
ZO-1	sc-33725	1: 200 (IF)	Mouse	Santa Cruz Biotechnology
Occludin	Ab216327	1:200 (IF)	Mouse	abcam

### Network pharmacological analysis

#### Target collection of naringin, osthole and colitis

The chemical structures and SMILES (simplified molecular input line entry specification) of naringin and osthole were retrieved from the TCMSP (Traditional Chinese Medicine Systems Pharmacology Database and Analysis Platform) (https://www.tcmsp-e.com/tcmsp.php) and PubChem databases (https://pubchem.ncbi.nlm.nih.gov/). Predicted targets of naringin and osthole were gathered from multiple sources, including the TCMSP, SwissTarget Prediction (http://www.swisstargetprediction.ch), PharmMapper (http://www.lilabecust.cn/pharmmapper/) and STITCH databases (https://ngdc.cncb.ac.cn/databasecommons/database/id/208). Disease targets were compiled from CTD (https://ctdbase.org/), DrugBank (http://www.drugbank.com), GeneCards (https://www.genecards.org/), OMIM (https://omim.org/), NCBI Gene (https://www.ncbi.nlm.nih.gov/gene), and DisGeNET (https://www.disgenet.org/) databases, focusing on colitis and ulcerative colitis-related keywords. Subsequently, duplicates among the targets of naringin, osthole, and colitis were eliminated, followed by the identification of the common targets through intersection analysis.

#### KEGG and GO enrichment analyses

To identify significantly enriched terms for biological processes (BP), cellular components (CC), molecular functions (MF), and KEGG pathways, the common target genes were subjected to enrichment analysis via the Database for Annotation, Visualization and Integrated Discovery (DAVID) (https://david.ncifcrf.gov/tools.jsp).

#### Protein–protein interaction (PPI) network and critical subnetwork

We first obtained a PPI network by inputting the shared targets of naringin, osthol, and colitis into the STRING database (https://cn.string-db.org/). We then visualized and analyzed this network in Cytoscape v. 3.9.1 (https://cytoscape.org/), where we performed a topology-based screening to identify the core targets.

#### Molecular docking

To identify the primary active compounds in important medicinal plants, we retrieved 3D structures from the PubChem database (http://www.ncbi.nlm.nih.gov/) and acquired the corresponding 3D structures of potential drug target proteins from the PDB database (http://www.rcsb.org/). Utilizing PyMOL 2.4.0, we conducted structural processing, followed by molecular docking employing AutoDock Vina. Evaluation of docking outcomes was based on free binding energy, where values below − 5.0 kJ/mol indicated a robust interaction between the compound and target protein. A reduced binding energy indicated a more stable docking complex, suggesting a heightened likelihood of interaction. The most favorable docking outcome, as indicated by the lowest free binding energy, was selected for presentation.

### Cell culture and treatment

The human colon cancer cell line HT-29 was obtained from the American Type Culture Collection (ATCC, USA) and were cultured in DMEM containing 100 IU/mL penicillin, 100 μg/mL streptomycin, and 10% fetal bovine serum (FBS) in a humidified incubator at 37 °C with 5% CO_2_. Based on the design of our in vivo experiments, this study extended the investigation to an in vitro setting to examine the effects of naringin and osthol on DSS-induced murine colitis. HT-29 cells were treated with 2 μg/mL LPS (E. coli 055: B5) with or without a 10 μM JNK inhibitor to induce inflammation. Subsequently, the cells were exposed to 25 μM naringin, 50 μM osthole, 25 μM naringin + 50 μM osthole, and 50 μM naringin + 50 μM osthole for 24 h. Cell samples were collected and stored at − 80 °C for further analysis. In this study all treatments were administered during the logarithmic growth phase of HT-29 cells, with phosphate buffered saline (PBS) used as the negative control.

### Cell viability and cytotoxicity assay

HT-29 cell viability and Cytotoxicity were assessed using a CCK-8 assay (Dojindo Laboratories, Tokyo, Japan) following the manufacturer's instructions. Cells were seeded in 96-well plates at a density of 3 × 10^4^ cells/mL, with 100 μL per well and five replicates per group. After cell adhesion, the appropriate drug concentration was added based on the groups. Cells were then cultured with 10 μL of CCK-8 per well at 37 °C for 1.5 to 2 h after 24 h of drug stimulation. Absorbance readings were taken at 450 nm using a microplate reader (Bio-Rad Instruments, Hercules, CA).

### Induction and therapies of colitis

The mice were housed under a 12-h light/12-h dark cycle for a one-week acclimatization period with free access to food and water. The animals utilized in this research are raised and managed in strict accordance with the Regulations on the Management of Experimental Animals and the guidelines formulated by the Experimental Animal Ethics Committee of Anhui Agricultural University. All animal experiments were carried out in strict accordance with the approval of Ethics Committee of Anhui Agricultural University (Approval No. AHAU2024053) on 3 May 2024. Male Kunming mice (6–8 weeks old) were bought from the Laboratory Animal Center of Anhui Medical University. Following a period of acclimatization spanning, the mice were randomly allocated to eight groups: (1) Normal control group, (2) DSS-induced colitis group, (3) N–L group (DSS + 25 mg/kg naringin), (4) N–H group (DSS + 50 mg/kg naringin), (5) O–L group (DSS + 25 mg/kg osthole), (6) O–H group (DSS + 50 mg/kg osthole), (7) N + O group (DSS + 50 mg/kg naringin + 50 mg/kg osthole), and (8) 5-ASA group (DSS + 100 mg/kg 5-ASA), with a sample size of 8 mice per group. Colitis was induced in mice as previously reported with slight modifications. Briefly, 3% DSS was administered orally to mice for 14 days to create the colitis model, and a blank control group was given the same dose of autoclaved water solution. From the induction of colitis until the conclusion of the experiment, a gavage was used to administer naringin and osthole (Shanghai Yuanye Bio-Technology Co. Ltd., Shanghai, China) to the mice. Both naringin and osthole were dissolved in a vehicle consisting of 10% DMSO and 90% olive oil. The two compounds were mixed in this common vehicle and co-administered to the mice in a single gavage. During the modeling and treatment period, mice were monitored daily for general health status. Body weight was recorded, and stool characteristics (including consistency and the presence of occult or gross blood) were assessed according to established criteria. These parameters were used to calculate the Disease Activity Index (DAI) daily. After 14 days, all mice were euthanized by cervical dislocation. The colon was excised, its length measured following a longitudinal incision, and tissue and fecal samples were collected for subsequent analysis.

### Hematoxylin and eosin staining and histopathologic analysis

Formalin-fixed, paraffin-embedded tissue sections were deparaffinized with xylene and graded ethanol, followed by staining with hematoxylin and eosin (H&E).

### Myeloperoxidase (MPO) assessment

The MPO activity in the sample was detected following the manufacturer's instructions using a kit (Nanjing Institute of Jianguo Bioengineering, Nanjing, China). The OD peak was measured at a wavelength of 460 nm utilizing an enzyme marker.

### Quantitative real-time PCR

HT-29 cells numbering 1 × 10^6^ were subjected to drug treatment for 24 h. Total RNA was extracted using TRIzol reagent (Biosharp), followed by reverse transcription of 1 µg of total RNA into cDNA using a cDNA Synthesis Kit (Vazyme). Quantitative PCR (qPCR) analysis was conducted with the MiniOpticon qPCR detection system (Bio-Rad Laboratories). Relative quantification was calculated using calculate the 2^−ΔΔCT^ method. Primer sequences can be found in Table 2.

**Table 2:** Oligonucleotide primers used for qPCR

IL-6	Forward	CTGGTCTTCTGGAGTACCATAGC
	Reverse	CTGGTCTTCTGGAGTACCATAGC
IL-1β	Forward	GAAATGCCACCTTTTGACAGTG
	Reverse	TGGATGCTCTCATCAGGACAG
TNF-α	Forward	GGAACACGTCGTGGGATAATG
	Reverse	GGCAGACTTTGGATGCTTCTT
IL-10	Forward	AGCCTTATCGGAAATGATCCAGT
	Reverse	GGCCTTGTAGACACCTTGGT
GAPDH	Forward	AATGGATTTGGACGCATTGGT
	Reverse	TTTGCACTGGTACGTGTTGAT

### Western blot

Proteins from the collected cells or colon tissues were extracted using RIPA lysis buffer containing PMSF. Protein samples were separated by SDS-PAGE and transferred onto polyvinylidene fluoride (PVDF) membranes. The membranes were blocked with 5% non-fat milk in PBST at room temperature for 2 h, followed by incubation with the corresponding primary antibodies overnight and subsequent incubation with HRP-conjugated secondary antibodies. Protein bands were visualized using an enhanced chemiluminescence (ECL) detection system.

### Immunofluorescence staining

HT-29 cells were cultured on glass coverslips and gently washed three times with PBS. The slides were then immersed in staining jars containing 4% paraformaldehyde and incubated at 4 °C for 15 min. After blocking with 10% bovine serum albumin (BSA) for 1 h, the samples were incubated with primary antibodies overnight at 4 °C, followed by incubation with secondary antibodies at room temperature for 2 h. Subsequently, nuclei were counterstained with DAPI for 10 min in the dark and mounted with an anti-fade reagent. Fluorescence images were captured using a fluorescence microscope (Nikon, Tokyo, Japan).

### 16S ribosomal RNA gene sequencing

The extraction of microbial DNA was performed using the HiPure Stool DNA extraction kit (Magen, Guangzhou, China) in accordance with the operating instructions. Subsequently, the PCR amplification was performed on the target region of the 16SrRNA gene. The amplicon was collected from a 2% agarose gel, purified with the AxyPrep DNA gel extraction kit (Axygen Biosciences, Union City, CA, USA) according to the manufacturer's instructions, and quantified with the ABI StepOnePlus real-time PCR system (Life Technologies, Foster City, USA). The purified amplicons were then subjected to double-ended sequencing (PE250) on the Illumina platform in accordance with standard procedures. Following the downloading of the sequencing data, species annotation is performed based on OTU/ASV sequences, and the abundance information of species at each level is statistically obtained based on OTU/ASV abundance information Subsequent bioinformatics analysis was then conducted on species composition, indicator species, alpha diversity, beta diversity and functional prediction, using OTU and species abundance tables.

### Metabolomics analysis

The extraction of samples was conducted using the homogenization lysis method, followed by separation through the utilization of an Agilent 1290 Infinity LC Ultra High-Performance Liquid Chromatography (UHPLC) HILIC column. Throughout the analysis process, the samples were placed in an automatic sampler maintained at 4 °C. To circumvent the impact of instrument detection signal fluctuations, a random sequence was utilized for uninterrupted sample analysis. The insertion of QC samples into the sample queue is a vital step for the monitoring and evaluation of system stability and the reliability of experimental data. The ABTripleTOF6600 mass spectrometer was then employed to collect primary and secondary spectra of the sample. The raw data was then converted by ProteoWizard (v3.0.6428) into MzML format, followed by peak alignment, retention time correction, and peak area extraction using XCMS program (online 3.7.1). For the data extracted by XCMS, metabolite structure identification and data preprocessing are first performed, followed by data analysis.

### Statistical analysis

Quantitative data were presented as mean ± SEM from a minimum of three biologically independent experiments or samples. Statistical analyses utilized GraphPad Prism 10 (GraphPad, San Diego, CA, USA), employing a two-tailed unpaired Student's t-test to evaluate differences between two groups. For comparisons among multiple groups one- or two-way analysis of variance (ANOVA) was conducted, followed by Dunnett's post hoc multiple comparison test. Statistical significance was defined as p < 0.05.

## Results

### Combination therapy of naringin and osthole can alleviate symptoms in DSS-induced acute colitis model in mice

**Fig. 1:**
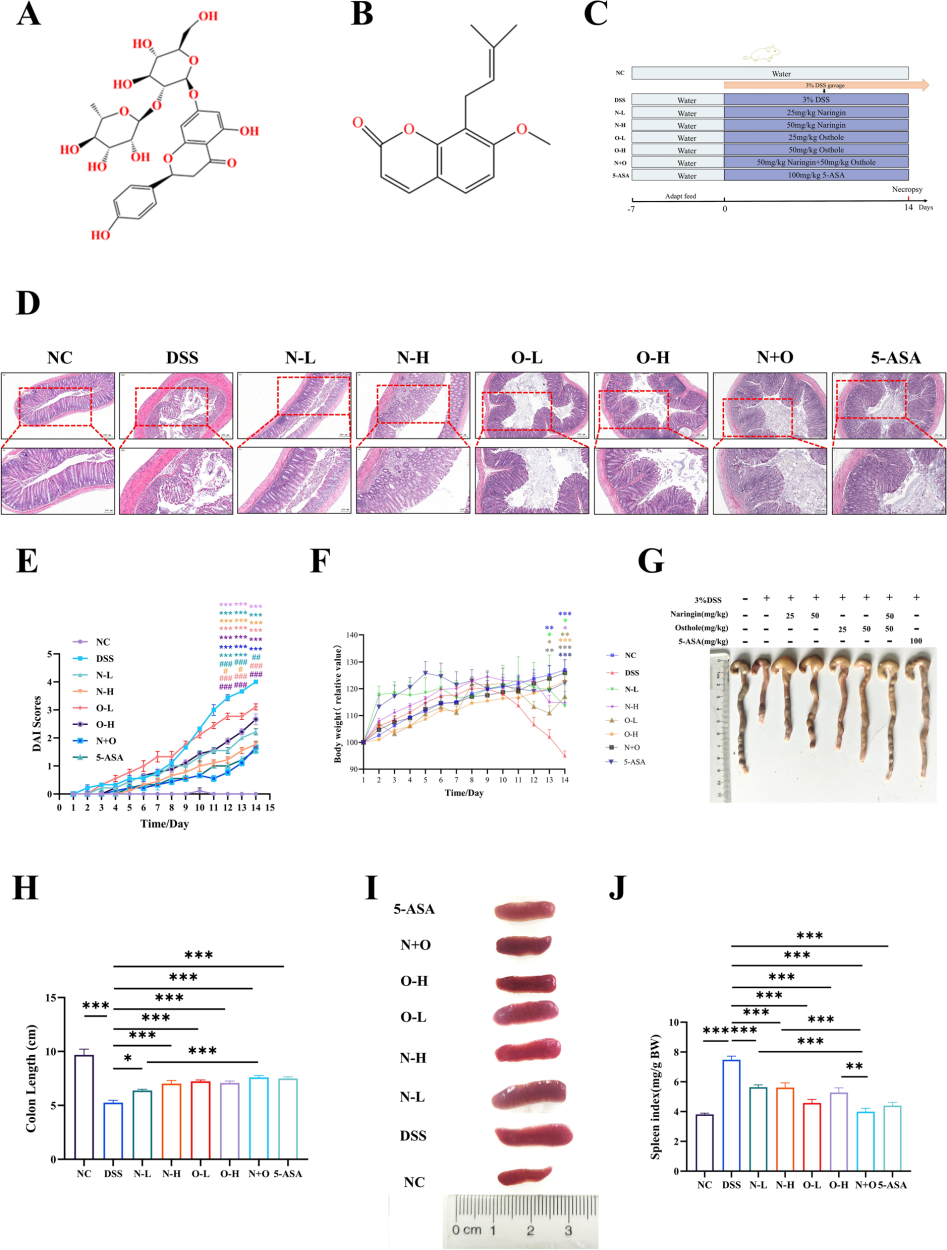
The effect of naringin and osthole alone or in combination on DSS-induced acute colitis model in mice. **A** Naringin chemical structure. **B** Osthole chemical structure. **C** Schematic diagram of animal experiment design. **D** Typical H&E staining of colon tissue sections, scale bar (Top: 200 µm; Bottom: 100 µm). **E**, **F** DAI scores and Weight changes. **G**, **H** Representative images of colon tissue and their length quantification. **I**, **J** Representative images and spleen index of the spleen. Data are presented as mean ± SEM (n = 12). *P < 0.05, **P < 0.01, ***P < 0.001 vs. DSS group; ^#^P < 0.05, ^##^p < 0.01, ^###^p < 0.001vs. N + O group

We have demonstrated the chemical structures of naringin and osthole (Fig. 1A and 1B). To evaluate the synergistic effects of naringin and osthol, we established a mouse model of acute colitis induced by DSS. (Fig. 1C). In the histological examination with H&E staining, mice in the DSS-treated group showed displayed colonic epithelial disorganization, disrupted crypt structure, and significant inflammatory infiltration in the lamina propria and submucosa. Notably, co-administration of naringin and osthole alleviated the above symptoms (Fig. 1D). Following induction of colitis with DSS, the mice exhibit hemorrhagic diarrhea, weight loss, and a shortening of colon length, and splenomegaly. These clinical manifestations are consistent with the established features of ulcerative colitis [21]. Activity Index (DAI) score markedly increased, aligning with established colitis indicators [22]. Our findings validate the enhanced effectiveness of combination therapy in facilitating weight gain, decreasing DAI scores, and mitigating splenomegaly (Fig. 1E–J).

### The combination therapy of osthole and naringin alleviates colitis in mice by repairing the intestinal barrier

**Fig. 2:**
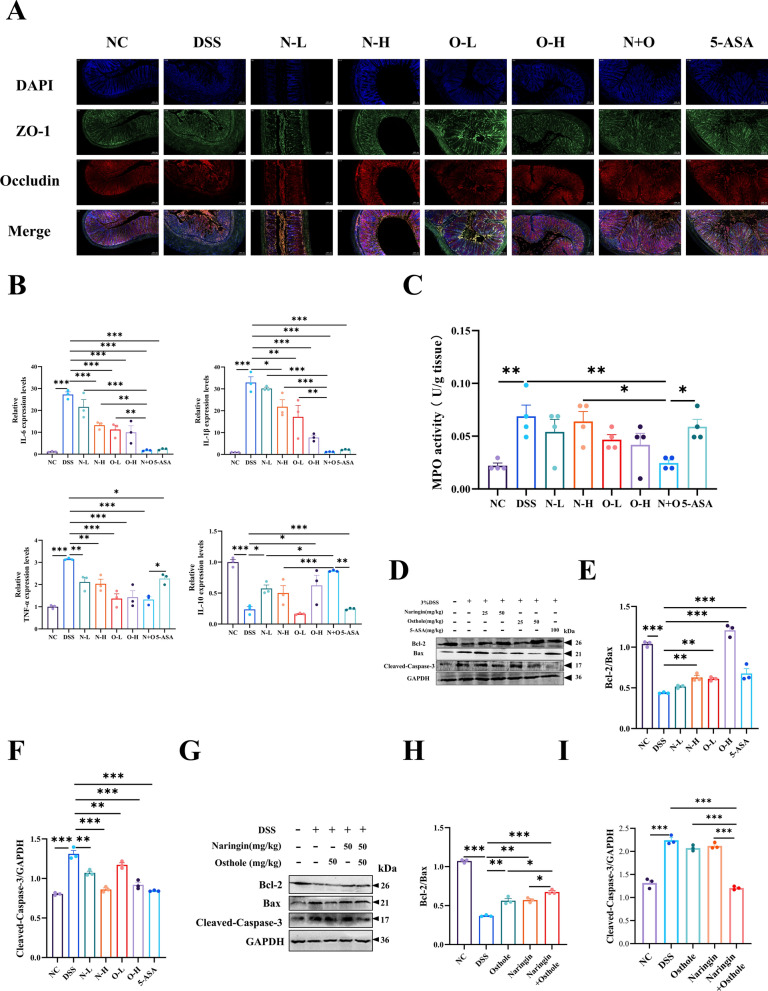
The effects of naringin and osthole alone or in combination on DSS-induced intestinal barrier. **A** Immunofluorescence staining analysis of ZO-1 (green) and Occludin (red) in colon tissue. Representative images are displayed. Scale bar = 100 µm. **B** RT‒qPCR for IL-6, IL-1β, TNF-α and IL-10 in colon sections. **C** Detection of myeloperoxidase activity (MPO). **D**–**I** Western blotting and quantitative analysis of apoptosis factors Bcl-2, Bax, and Cleaved-Caspase-3. Data are presented as mean ± SEM (n = 3). *P < 0.05, **P < 0.01, ***P < 0.001, ****P < 0.0001

ZO-1 and occludin are crucial tight junction proteins, with their expression serving as indicators of intestinal mucosal barrier integrity [23]. The study results show that after DSS stimulation, the expression of ZO-1 and occludin is significantly reduced, and subsequently restored through single or combined treatment, especially in the group treated with naringin and osthole (Fig. 2A). Previous studies have demonstrated that treatment with DSS leads to elevated levels of IL-1β, IL-6, and Tumor Necrosis Factor-α (TNF-α) [24]. In our study, treatment with drugs resulted in the downregulation of pro-inflammatory cytokines (IL-6, IL-1β, and TNF-α) in colon tissue, along with an upregulation of the anti-inflammatory cytokine IL-10 (Fig. 2B). The combination treatment exhibited the most pronounced effect, and a comparable pattern was observed in bone marrow myeloperoxidase levels (Fig. 2C). Additionally, ulcerative colitis is characterized by heightened apoptosis of intestinal epithelial cells [25]. Western blot analysis indicated that high doses of naringin and osthole most effectively upregulated Bcl-2/Bax expression and downregulated cleaved- caspase-3, which guided our choice of the high-dose groups for subsequent experiments (Fig. 2D–I). These findings suggest that the combination of naringin and osthole effectively promotes intestinal barrier repair by reversing the expression of tight junction proteins, reducing the release of inflammatory cytokines, and attenuating of intestinal epithelial cells.

### The combined administration of naringin and osthole can rescue the reduction of abundance in the gut microbiota

**Fig. 3:**
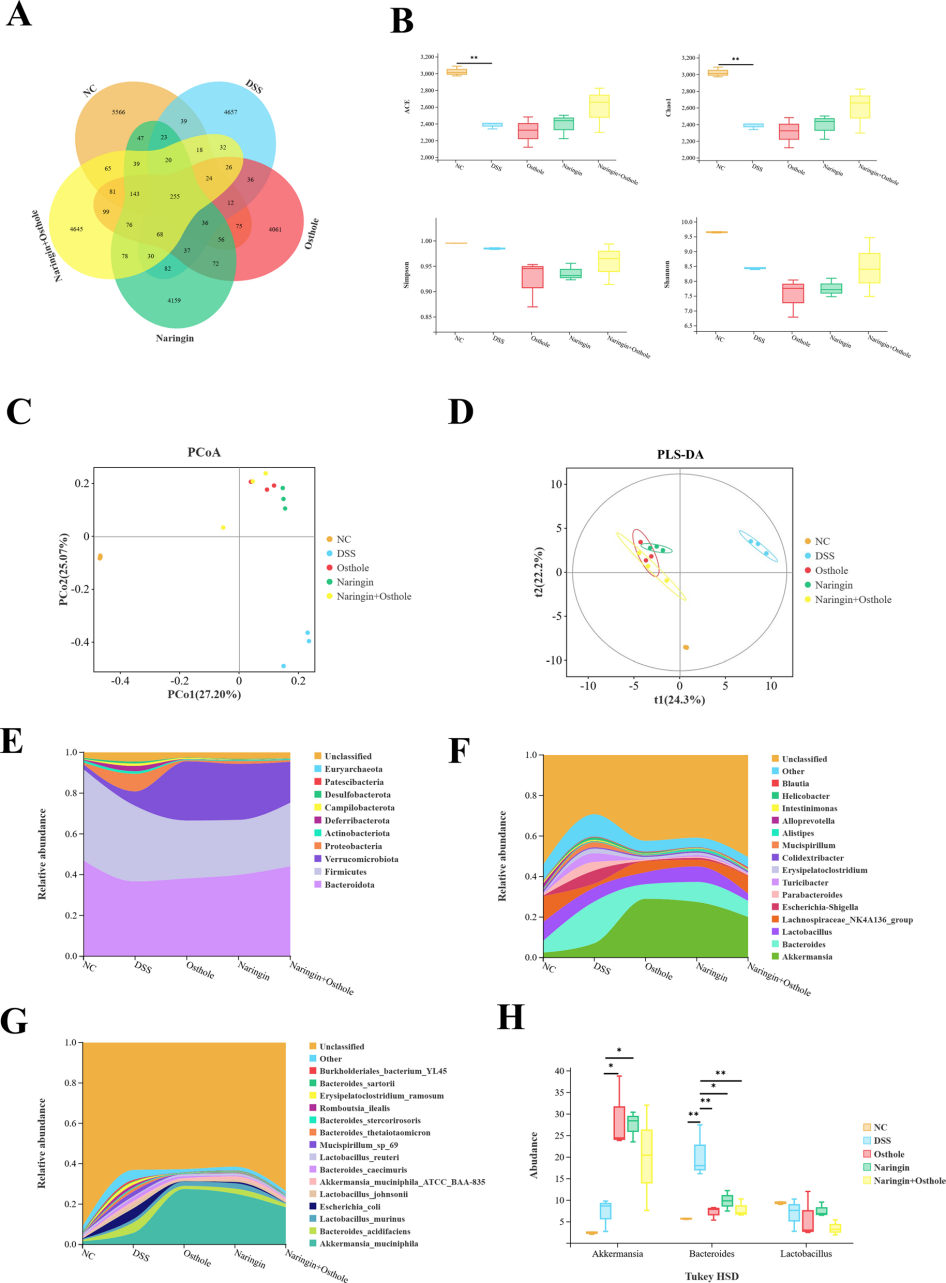
The combined administration of naringin and osthole can rescue the reduction of abundance in the gut microbiota. **A** The Venn diagram shows a comparison of five common species. **B** α-diversity indices. **C**, **D** PCoA and PLS-DA analysis. **E**–**G** Analysis of species composition at the phylum, species, and genus levels in five sample groups. **H** Three groups of indicator bacteria were screened at the species level using Tukey HSD assay. The default threshold is p-value < 0.05. Data are presented as mean ± SEM (n = 3). *P < 0.05, **P < 0.01

To comprehensively evaluate the effects of naringin and osthole on the gut microbiota, 16S rRNA sequencing was employed for analysis. We identified 255 common microbial species across the five sample groups (Fig. 3A). The ACE, Chao1, Simpson, and Shannon indices indicated that the α-diversity of the gut microbiota increased following treatment with naringin and osthole (Fig. 3B). This indicates an enhancement in gut microbiota diversity following administration. PCoA and PLS-DA analyses revealed distinct intra- and inter-group differences in the microbial communities (Fig. 3C and 3D). Analysis revealed a decrease in Bacteroidetes and Firmicutes, and an increase in Proteobacteria and Verrucomicrobiota at the phylum level in the DSS treatment group, consistent with prior studies [26]. In contrast, the groups receiving either monotherapy or combination therapy exhibited the opposite results (Fig. 3E). At the genus and species level, the gut microbiota of mice is predominantly comprised of Akkermansia, Bacteroides, Lactobacillus and Escherichia coli (Fig. 3F and 3G). Subsequently, the significant differences in the impact of each indicator species will be evaluated using Tukey HSD. At the genus level, Akkermansia, Bacteroides, and Lactobacillus emerge as the most influential strains (Fig. 3H). In summary, the study demonstrates that the combined administration of naringin and osthole promotes gut microbial homeostasis and enhances the α-diversity of the microbiota.

### Combination therapy of naringin and osthole regulates the levels of microbial tryptophan metabolites

**Fig. 4:**
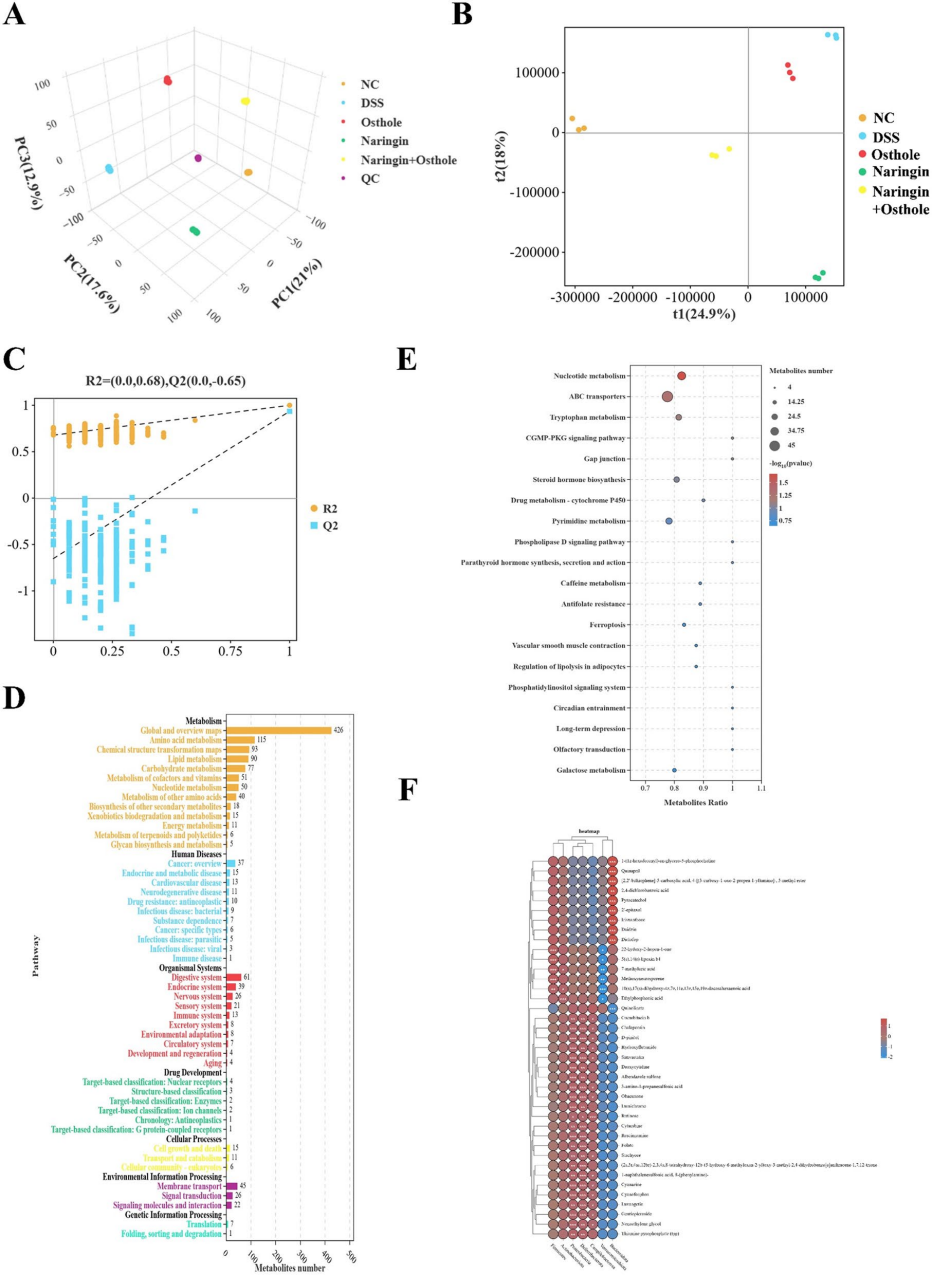
The combination therapy of naringin and osthol restored metabolic disorders under colitis. **A**, **B** PCA and PLS-DA analysis. **C** Permutation test is used to evaluate the accuracy of PLS-DA. **D** Statistics of KEGG enrichment numbers of differential metabolites. **E** The top 20 significantly differentially expressed metabolite pathways enriched by KEGG. **F** Combined Persion Analysis of Microbial Communities and Metabolites. The red dots indicate positive correlation, while the blue dots indicate negative correlation. The intensity of color is directly proportional to the intensity of Persion correlation. Data are presented as mean ± SEM (n = 3). *P < 0.05, **P < 0.01, ***P < 0.001

Changes in metabolic levels are crucial indicators for evaluating intestinal diseases [27]. We employed non-targeted metabolomics to investigate potential alterations in metabolites and metabolic pathways. 3D-PCA and PLS-DA analyses indicated significant differences between the DSS-treated group and the treatment groups (Fig. 4A and 4B). The accuracy of PLS-DA was assessed using a permutation test (Fig. 4C). Subsequently, the metabolite set was classified according to various criteria including metabolism, human diseases, organic systems, drug development, environmental information processing, cellular processes, and genetic information processing (Fig. 4D). Notably, the metabolic classification revealed a significant enrichment of 115 amino acid metabolites (Fig. 4D). Among the enriched metabolic pathways, amino acid tryptophan metabolism stood out prominently (Fig. 4E). Subsequently, Pearson correlation analysis revealed a strong negative correlation between quinolinate and the phylum Bacteroidetes (Fig. 4F). Quinolinate is a key metabolite in the kynurenine pathway of tryptophan catabolism breakdown [28]. Therefore, we hypothesize that the combined administration of naringin and osthole primarily enhances intestinal barrier repair by modulating the abundance of Bacteroidetes and reducing the levels of the downregulated tryptophan metabolite quinolinic acid.

### Combination therapy of naringin and osthole inhibits inflammatory damage in HT-29 cells in vitro

**Fig. 5:**
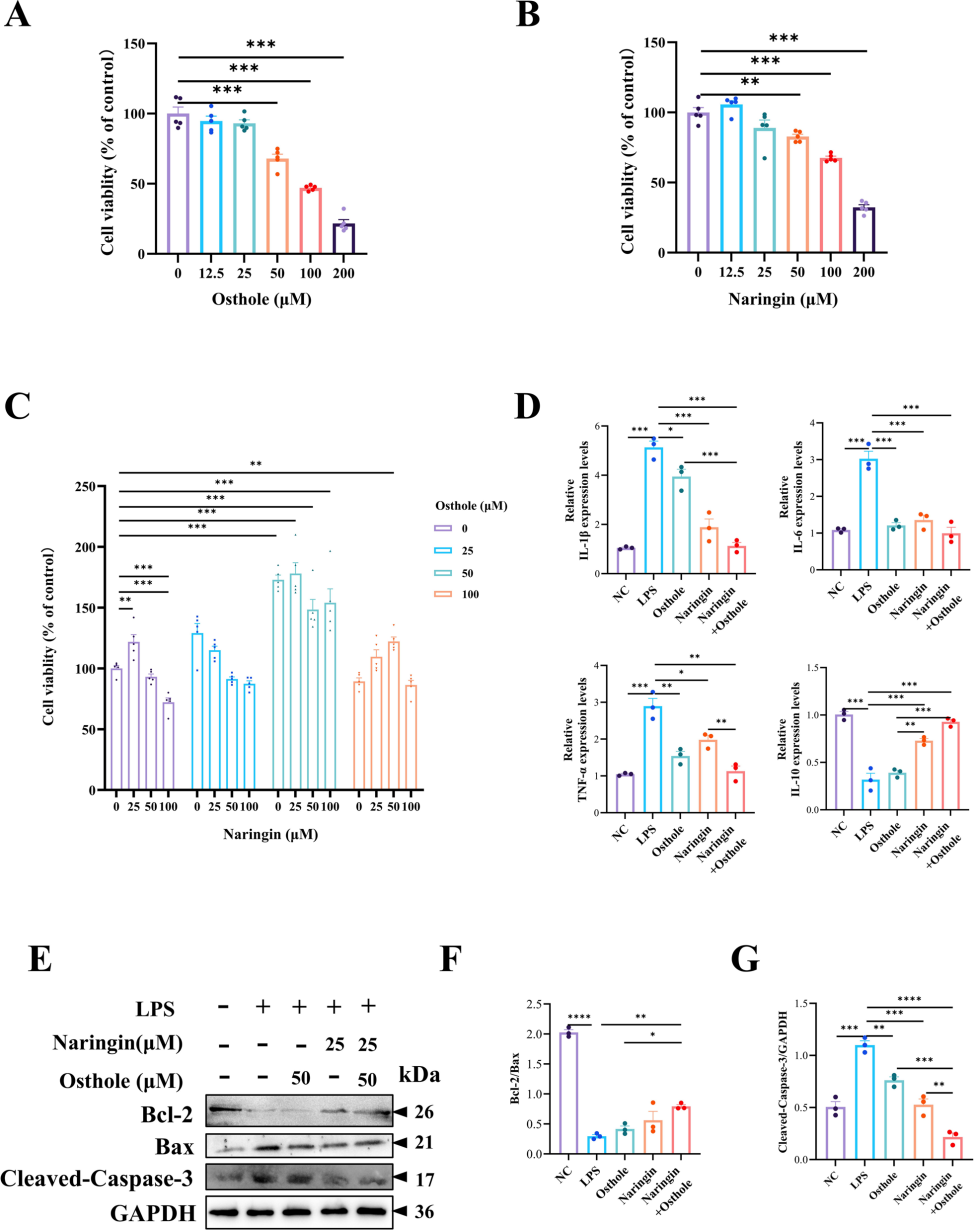
Combination therapy of naringin and osthole inhibits inflammatory damage in vitro. **A**–**C** CCK-8 detection of cell viability. **D** Perform RT-qPCR on IL-6, IL-1β, TNF-α, and IL-10 in HT-29 cells. **E**–**G** Western blotting and quantitative analysis of apoptosis factors Bcl-2, Bax, and Cleaved-Caspase-3. Data are presented as mean ± SEM (n = 3). *P < 0.05, **P < 0.01, ***P < 0.001, ****P < 0.0001

Previously, we evaluated the therapeutic effects of the combined administration in vivo. To assess its efficacy in vitro, we established an inflammation-induced injury model in HT-29 cells through external stimulation [29]. Cell viability of HT-29 cells was assessed using the CCK-8 assay, which showed a dose-dependent decrease when naringin or osthole exceeded 50 μM. Notably, the combination of 25 μM naringin and 50 μM osthole exerted the most significant protective effect against inflammatory injury in HT-29 cells. Consistent results were also observed in the expression of inflammatory markers (IL-6, IL-1β, TNF-α, and IL-10) and in the findings of Western blot analysis (Fig. 5A–G).

### Prediction based on network pharmacology: Naringin and osthole may alleviate ulcerative colitis through JNK/NF-κB signaling pathways in combination

**Fig. 6:**
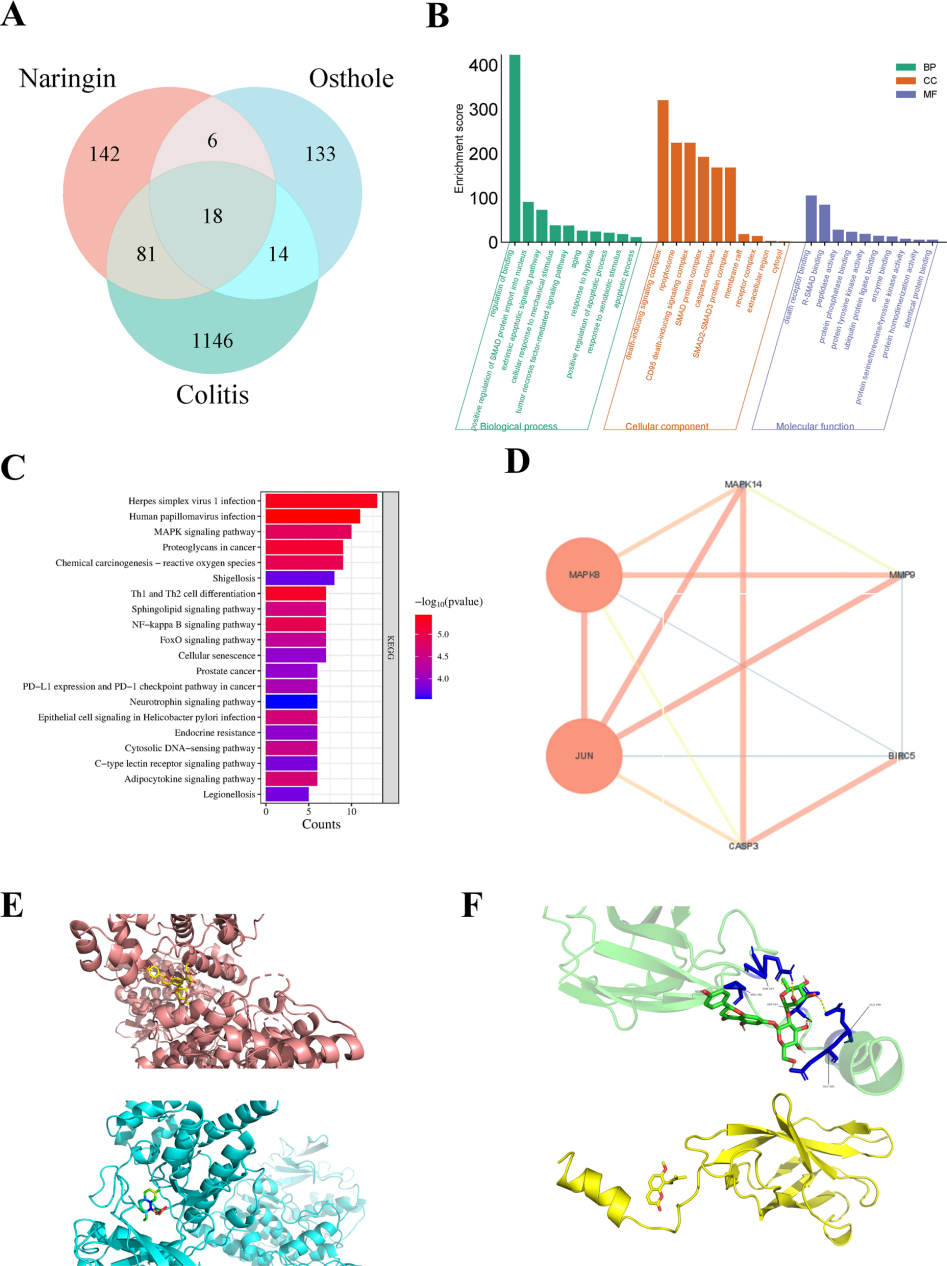
Prediction based on network pharmacology: Naringin and osthole may alleviate ulcerative colitis through JNK/NF-κB signaling pathways in combination. **A** Venn diagram of target genes for naringin, osthole and colitis. **B** GO enrichment analysis for key targets (top 10 were listed). **C** KEGG pathway enrichment analysis (top 20 were listed), the abscissa label represents enrichment counts of pathways. **D** PPI network processed by Cytoscape v.3.9.1 website. **E** Molecular docking modeling of naringin (top) and osthole (bottom) targeting JNK, respectively. **F** Molecular docking modeling of naringin (top) and osthole (bottom) targeting NF-κB respectively

To predict the potential targets of naringin and osthole in colitis, we conducted a comprehensive search and screening of their respective targets. By constructing a Venn diagram of the target sets, 18 common targets were identified (Fig. 6A). The DAVID annotation tool was then utilized for GO and KEGG enrichment analyses. GO enrichment analysis comprises three main categories: Biological Process (BP), Cellular Component (CC), and Molecular Function (MF). KEGG enrichment analysis identified the MAPK pathways and NF-κB pathways as two key signaling pathways (Fig. 6B and 6C). The protein–protein interaction (PPI) network analysis highlighted several key targets, including MAPK8, JUN, MAPK14, CASP3, MMP9, and BIRC5. Among them, MAPK8 and JUN exhibited larger node sizes and stronger connectivity (Fig. 6D). Subsequently, we performed molecular docking of naringin and osthole with JNK and NF-κB. The results showed that the binding energies of the two compounds with JNK were − 7.4 kcal/mol and − 9.5 kcal/mol, respectively, while their binding energies with NF-κB were − 7.2 kcal/mol and − 5.5 kcal/mol, respectively. These findings indicate that both naringin and osthole exhibit favorable binding affinities toward JNK and NF-κB proteins (Fig. 6E and 6F).

### The combined administration of naringin and osthole alleviates colitis in mice by inhibiting the JNK/NF-κB signaling pathway in vitro and in vivo

**Fig. 7:**
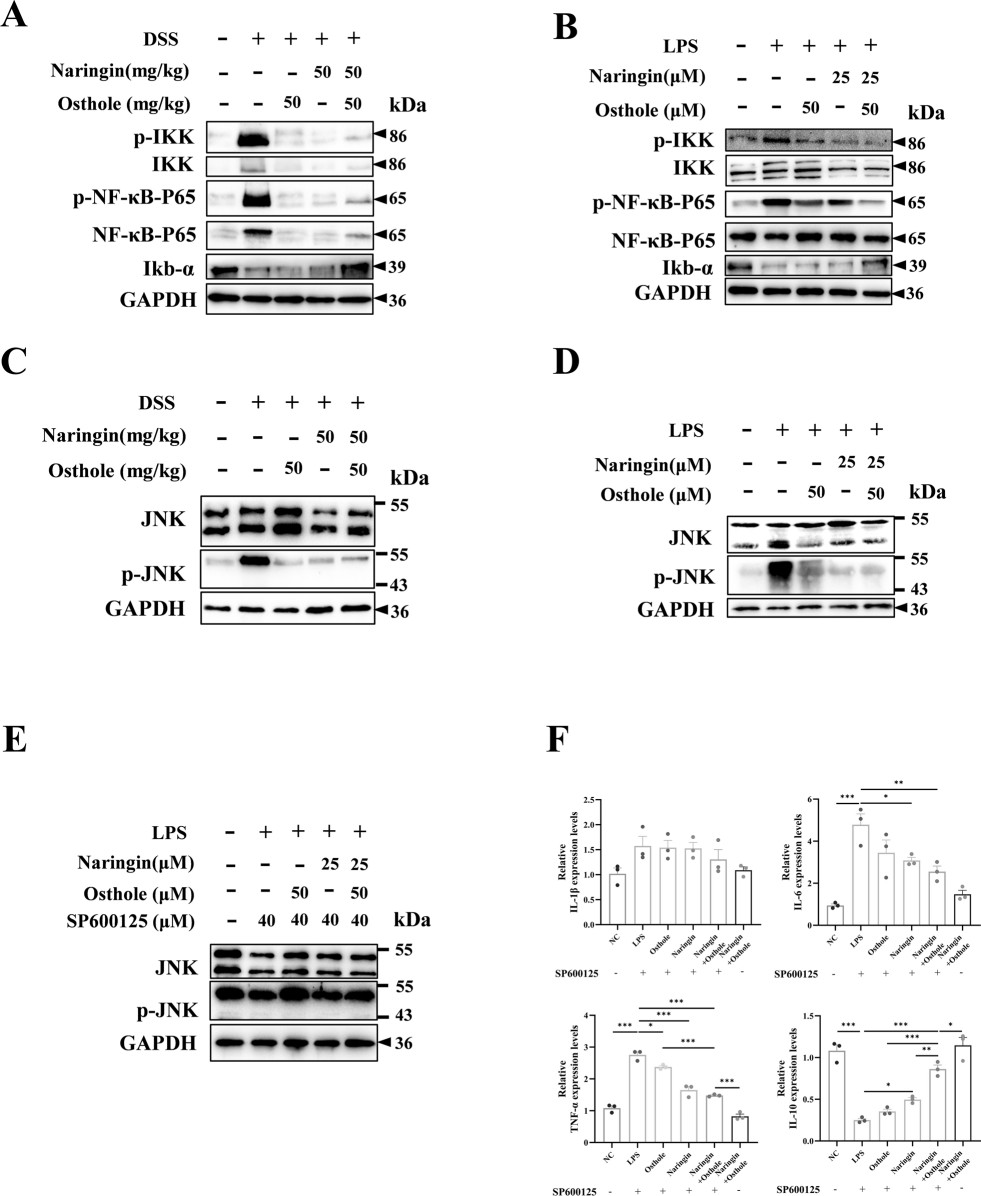
The combined administration of naringin and osthole alleviates colitis in mice by inhibiting the JNK/NF-κB signaling pathway in vitro and in vivo. **A**, **B** Western blot for NF-κB signing pathway in vivo and in vitro. **C**, **D** Western blot for JNK signing pathway in vivo and in vitro. **E** Western blot of JNK signing pathway in vitro after JNK inhibitor (SP600125) treatment. Data are presented as mean ± SEM (n = 3). **F** RT-qPCR of IL-6, IL-1β, TNF-α and IL-10 in HT-29 cells after pretreatment with JNK inhibitor SP600125. Data are presented as mean ± SEM (n = 3). *P < 0.05, **P < 0.01, ***P < 0.001

Emerging evidence suggests that bioactive compounds, particularly polyphenols found in fruits and vegetables, can modulate key inflammatory pathways, including NF-κB and JAK/STAT [30]. Network pharmacology analyses have further identified the convergence of MAPK8 and JUN as a critical target within JNK signaling pathways [31]. Protein expression levels of proteins associated with the JNK/NF-κB signaling cascade were evaluated using Western blot analysis. Consistent with expectations, naringin and osthole, administered either individually or in combination, effectively inhibited the phosphorylation and expression of proteins within the NF-κB signaling pathway, with the combination treatment exhibiting the most pronounced inhibitory effect (Fig. 7A and 7B). We also observed activation of the JNK subfamily within the MAPK pathway, which was consistent with the results from the NF-κB protein Western blot analysis (Fig. 7C and 7D). Subsequent cellular experiments using the JNK inhibitor (SP600125) in combination with LPS further supported these findings, demonstrating that the combined treatment with naringin and osthole restored the downregulated expression of JNK proteins (Fig. 7E). Notably, prior RT-qPCR experiments ruled out any direct impact of the inhibitors on the expression of inflammatory factors (Fig. 7F). In conclusion, naringin and osthole act synergistically to enhance pharmacological efficacy against colitis in mice through modulation of the JNK/NF-κB signaling pathway.

## Discussion

**Fig. 8:**
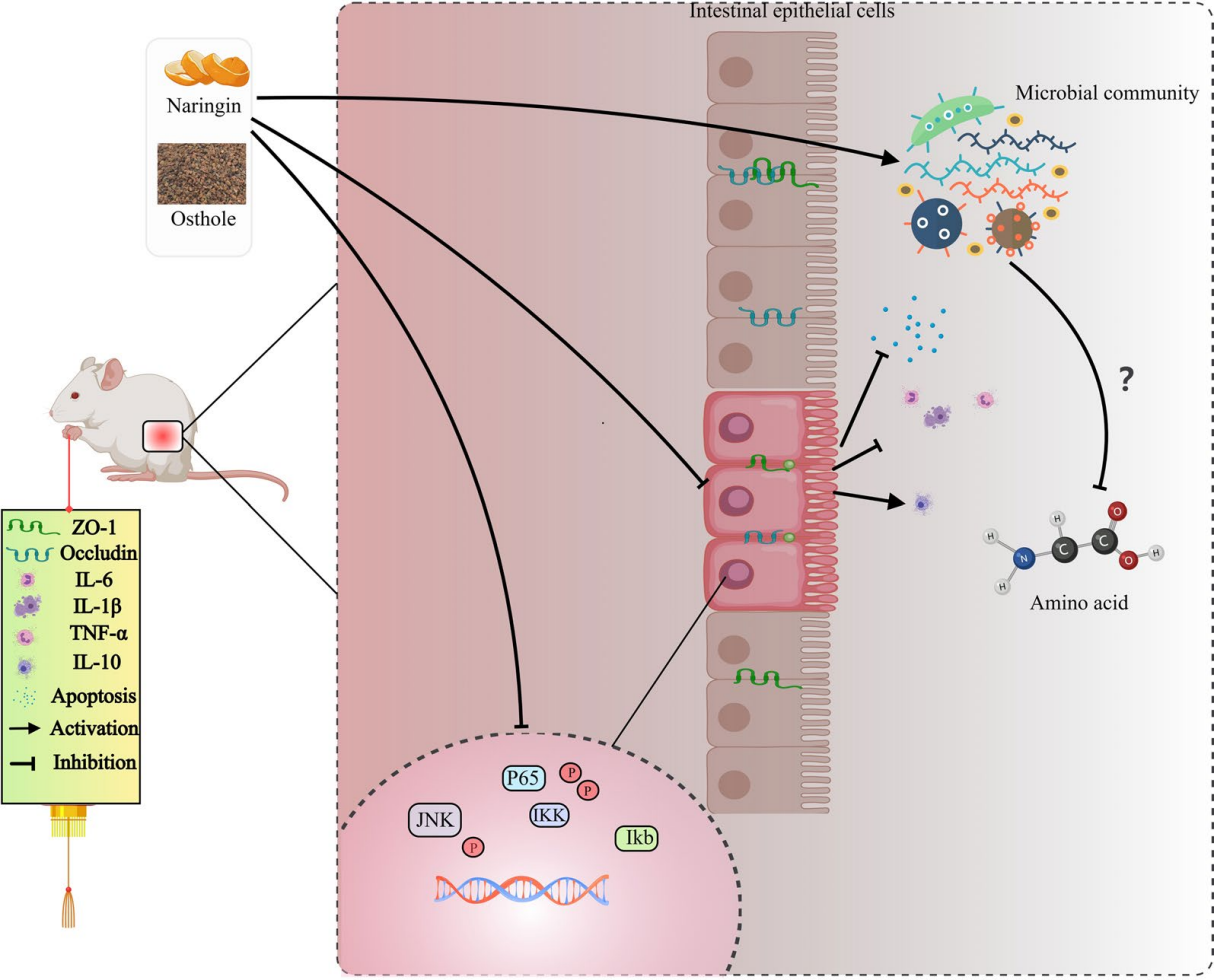
The mechanism by which naringin combined with osthole improves DSS-induced chronic colitis

Emerging evidence suggests that patients diagnosed with UC experience diminished overall survival and an increased risk of colorectal cancer [32]. While 5-aminosalicylic acid remains a mainstay treatment for mild to moderate UC, advanced therapies directed against specific inflammatory pathways are necessary for moderate to severe cases [33, 34]. Our investigation reveals that the combination of naringin and osthole exhibits superior efficacy in reducing inflammatory damage both in vivo and in vitro. Mechanistically, this combination therapy appears to suppress tryptophan conversion to quinolinate by decreasing Bacteroidetes abundance, ultimately attenuating inflammation through the JNK/NF-κB signaling pathway (Fig. 8).

In our prior research, we characterize the anti-inflammatory activity of naringin in endometritis [16]. Osthole, a bioactive constituent derived from medicinal flora including *Cnidium monnieri* and *Angelica pubescens*, displays multifunctional pharmacological properties encompassing antitumor, anti-inflammatory, neuroprotective, osteogenic, and cardioprotective activities [35]. Emerging studies indicate that osthole suppresses the activation of the P38 MAPK signaling pathway and reduce the production of IL-6 and TNF-α in LPS-induced macrophages. Furthermore, osthole has been found to be pivotal in managing neuropathic pain by remodeling gut microbiota subsequent metabolite regulation [15, 36]. Nonetheless, the exact molecular basis through which osthole’s efficacy in treating UC by modulating the interplay among microbiota, microbiota metabolites, and epithelial mechanical barriers remains elusive, particularly regarding its synergistic mechanisms when co-administered with naringin. The gut microbiota, frequently termed the "second genome", critically regulates fundamental physiological processes—including host immunity and metabolism through persistent exposure to microbial metabolites [37]. Extensive research has linked disruptions in gut microbiota and metabolism to UC [38, 39]. Our study, consistent with prior evidence, confirms that DSS-induced colitis in mice drives significant dysbiosis in various microbial communities, predominantly Bacteroidetes, Firmicutes, Proteobacteria, and Verrucomicrobiota. Notably, the ratio of Firmicutes to Bacteroidetes emerges as a potential predictor of UC [40]. Disrupted gut microbial homeostasis precipitates deterioration of mucosal barrier function, manifested through dysregulated localization of tight junction markers ZO-1 and occludin in our immunofluorescence staining. The integrity of the epithelial barrier normally confines the microbial community to the intestine; however, disruption of this barrier allows microorganisms and their metabolites to breach the intestinal barrier and accumulate in the host's circulatory system [8]. The DSS group showed a decrease in the abundance of Bacteroidetes and Firmicutes, as confirmed by 16S ribosomal RNA gene sequencing, consistent with prior studies [41]. Significantly, the combinatorial regimen exerted superior modulatory effects on reducing Bacteroidetes abundance versus individual naringin and osthole administration. Consequently, targeting microbiota-regulated metabolites to restore intestinal barrier integrity could present a promising strategy for treating IBD [42].

Metabolic disturbance can lead to nutrient deprivation in intestinal epithelial cells and immune dysfunction, thereby disrupting intestinal barrier integrity and exacerbating the progression of UC [43]. Our results establish the combination of naringin and osthole substantially potentiated the amino acid metabolite pathway. Further experimental analyses elucidated the role of tryptophan metabolism among the top 20 differentially altered metabolic pathways. Tryptophan can undergo various metabolic pathways through host canine urea and microbial indole pathways [44]. Quinolinate, a critical metabolite in the primary tryptophan metabolism pathway (the kynurenine pathway), serves as an indispensable regulator in this pathway [45]. As a precursor of NAD^+^, quinolinate can redirect a portion of tryptophan catabolism to boost cellular NAD^+^ levels in response to inflammation, injury and infection [28]. Certain cells have an increased demand for NAD^+^ during inflammation, injury and infection [46, 47]. Integrated microbiota-metabolome analysis revealed a significant negative correlation between Bacteroidetes abundance and quinolinate concentrations following the concomitant use of naringin and osthole. Collectively, these data mechanistically imply that the combined treatment may not only increase Bacteroidetes abundance but also enhance quinolinate involvement in directing tryptophan metabolism to potentiate its therapeutic efficacy against systemic inflammation [48].

Jun N-terminal kinase (JNK) is apivotal regulatory protein in the mitogen-activated protein kinase (MAPK) signaling pathway [49]. Despite extensive research on the JNK pathway, further investigation is warranted due to its intricate crosstalk within the multiprotein interactome necessitates deeper mechanistic interrogation. Over the past decade, the advent of systems biology, metabolomics, and allied disciplines has driven the development of a promising approach in drug discovery known as network pharmacology [50]. In this study, network pharmacology identified MAPK8 and JUN as crucial targets. JUN, a substrate of JNK, is a critical factor of nuclear transcription [51]. MAPK8(JNK1) is implicated in the JNK1/c-Jun signaling pathway, which is crucial in chronic pain pathogenesis [52]. Western blotting analysis revealed that the combination of naringin and osthole effectively inhibits the JNK signaling pathway both in vitro and in vivo. However, in vitro studies showed only partial symptom relief when the combination was used with JNK inhibitors, likely due to incomplete JNK pathway inhibition by the inhibitors.

Building upon these findings, we posit that the combined administration of naringin and osthole holds significant disease-modifying potential. Notwithstanding the therapeutic potential demonstrated herein, two mechanistic lacunae merit emphasis. While untargeted metabolomics provides comprehensive understanding of metabolic pathways and compounds, targeted metabolomics studies are imperative to validate and quantify changes in pivotal metabolites. Furthermore, additional research is needed to delineate the effects of naringin and osthole on specific microorganisms and metabolic pathways.

## Conclusion

In summary, our study indicates that the combined administration of naringin and osthole effectively mitigates symptoms of DSS-induced colitis to a greater extent than their individual administration. This enhanced efficacy is attributed to various mechanisms, such as the modulation of intestinal microbiota and tryptophan metabolites, restoration of intestinal barriers, and reduction of apoptosis. These findings lay a solid foundation for colitis treatment and underscore the potential of combination therapy as a viable treatment approach. Future research should focus on elucidating the specific mechanisms through which combined osthole and naringin formulations interact within the microbial flora-amino acid metabolite pathway.

## Data Availability

All other data that support the findings of this study are available on request from the corresponding author.
